# Identification of immune-associated biomarkers in diagnosing allergic rhinitis^☆^

**DOI:** 10.1016/j.waojou.2025.101084

**Published:** 2025-07-28

**Authors:** Qi Cheng, Jie Fei, Yuanfen Liao, Hang Lin, Yubao Cui

**Affiliations:** a Clinical Research Center, The Affiliated Wuxi People's Hospital of Nanjing Medical University, Wuxi 214023, China; b Department of Otolaryngology, The Affiliated Wuxi People's Hospital of Nanjing Medical University, Wuxi 214023, China; c Department of Allergy, The Affiliated Hospital of Qingdao University, Qingdao 266003, China

**Keywords:** Allergic rhinitis, Machine learning algorithms, Biomarkers, Immune infiltration

## Abstract

**Background:**

Allergic rhinitis (AR), as a prevailing disease worldwide, is a common chronic inflammatory disease of the upper airway; however, its prognosis did not meet expectations of the general public. Regarding the importance of treatment for AR, biomarkers with high sensitivity and precision are in urgent need.

**Methods:**

To detect the potential mechanisms associated with the AR pathology, the differentially expressed gene data from the clinical dataset GSE19187 and GSE46171 were used for weighted gene co-expression network analysis. Additionally, 5 machine-learning algorithms were integrated to screen for novel diagnostic biomarkers for AR.

**Results:**

This analysis identified 133 genes enriched in the black and purple module pathways from weighted gene co-expression network analysis (WGCNA). The 4 key diagnostic genes—CST1, CST2, SERPINB4, and TOX—were identified through machine learning algorithms and validated for their diagnostic potential with high area under the curve (AUC) values in external datasets. Immune infiltration analysis indicated a higher proportion of resting mast cells in AR samples compared to controls, with CST1, CST2, and SERPINB4 showing high expression in these cells. Real-time PCR validation confirmed that CST1, CST2, and SERPINB4 were upregulated in AR samples while TOX was downregulated.

**Conclusion:**

Our study identifies critical molecular markers and immune cell alterations associated with AR, providing insights into its pathogenesis and potential diagnostic biomarkers for clinical application. Future research should focus on validating these findings in larger cohorts to facilitate the development of targeted therapies for AR.

## Introduction

Allergic rhinitis (AR) is a common chronic inflammatory disorder that affects millions of individuals worldwide and has become a significant global health concern.^1^ It is primarily driven by atopic sensitization, an immunoglobulin E (IgE)-mediated immune response to environmental allergens, and manifests with symptoms such as nasal itching, sneezing, rhinorrhea, and nasal obstruction across all age groups.^2^ Although considerable progress has been made in understanding the clinical features of AR, the underlying molecular mechanisms that contribute to nasal mucosal inflammation and remodeling remain poorly understood.^3^ A deeper exploration of these mechanisms may provide new insights into the development of more effective therapeutic strategies for AR. Previous studies have identified several genes—such as *TLR4*, *CD14*, *STAT1*, *CST1*, and *CLC*—as being implicated in the susceptibility to AR and as potential biomarkers for disease development.^4^^,^^5^ However, these findings alone do not fully explain the high prevalence and complex pathogenesis of AR, highlighting the need for further investigation.

Recent studies suggest that the nasal epithelium plays a crucial role in the pathogenesis of AR, acting as the first line of defense against inhaled allergens and orchestrating downstream immune responses.^6^^,^^7^ To explore the molecular underpinnings of AR, transcriptomic approaches such as microarray analysis have proven valuable for profiling gene expression patterns. While RNA sequencing is a newer and more expensive technology, microarrays remain widely used due to their cost-effectiveness, reproducibility, and abundance of publicly available datasets.^8^ Using microarray data from the Gene Expression Omnibus (GEO), researchers can systematically identify differentially expressed genes (DEGs) between allergic patients and healthy controls, revealing key inflammatory pathways like JAK-STAT, NF-κB, and toll-like receptor signaling.^9^, ^10^, ^11^, ^12^, ^13^

In addition to epithelial cells, immune cells including eosinophils, mast cells, and dendritic cells contribute to AR pathogenesis. For example, epithelial-derived cytokines such as IL-25 and IL-33 promote Th2 immune responses, while mast cells play a central role in acute allergic reactions by releasing inflammatory mediators upon antigen re-exposure.^14^

Leveraging advances in bioinformatics and machine learning, this study aimed to identify novel gene targets in AR by analyzing microarray datasets. We performed differential expression analysis on dataset GSE19187, followed by weighted gene co-expression network analysis (WGCNA) to detect modules associated with AR. Cross-referencing WGCNA results with gene ontology (GO) and KEGG pathway enrichment highlighted 81 crosstalk genes strongly related to AR. Subsequently, 5 machine learning algorithms—LASSO, SVM-RFE, random forest, XGBoost, and Boruta—were applied to prioritize hub genes. Four intersecting genes—CST1, CST2, SERPINB4, and TOX—were identified, and their expression was validated using dataset GSE46171 and human NP69 cells stimulated with house dust mite extract. Our findings suggest these genes play key roles in AR pathogenesis and warrant further validation in larger cohorts.

## Materials and methods

### Microarray data

**Fig. 1:**
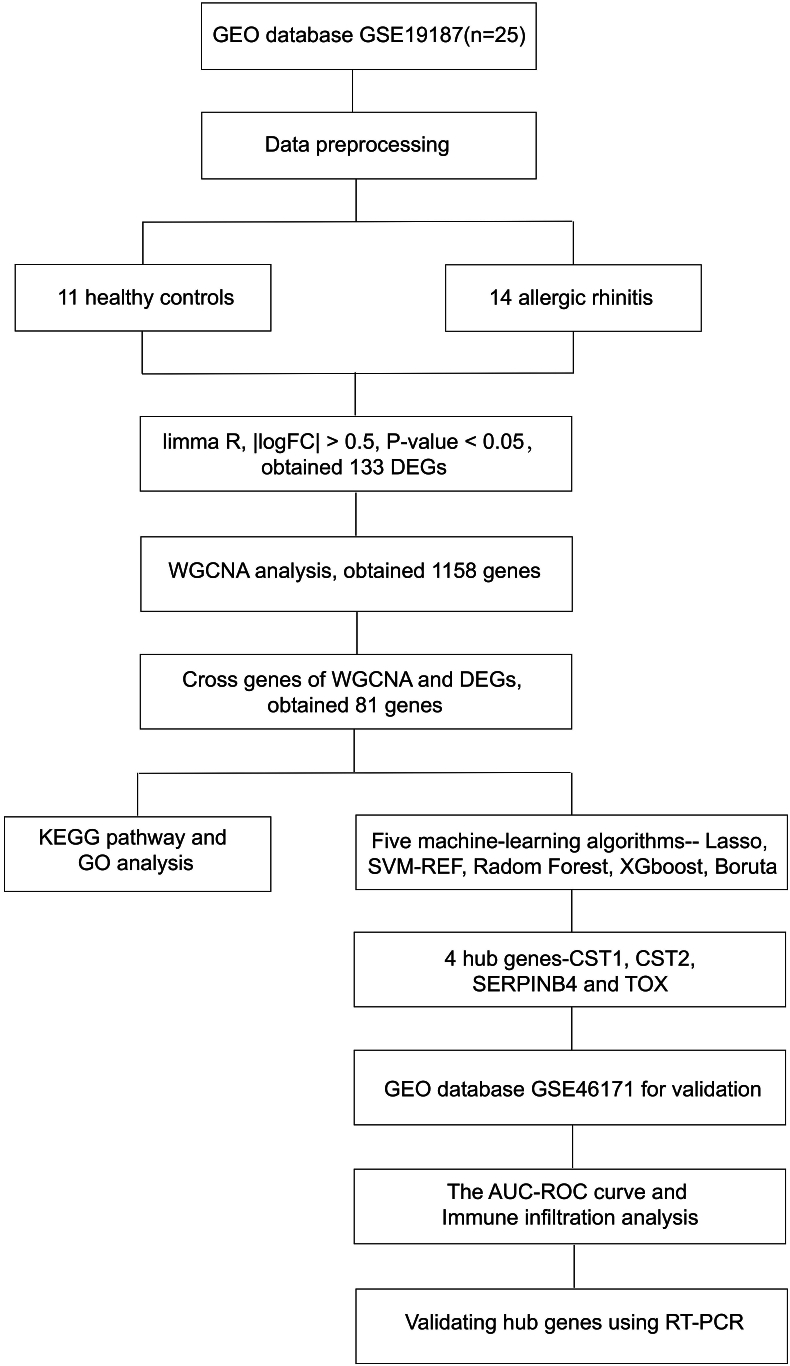
**Workflow diagram of data collection and analysis**.

Allergic rhinitis (AR)-related expression datasets (GSE19187^15^ and GSE46171^16^) were obtained through the GEO database (http://www.ncbi.nlm.nih.gov/geo/). Besides, the GSE19187 dataset, involving collected nasal epithelium samples from 11 control subjects and 14 subjects with rhinitis, was used to recognize DEGs and the mechanisms of asthma and AR.^17^ This study explored the detailed mechanism of rhinitis utilizing nasal epithelia expression profiles. Nasal epithelial samples from GSE46171 were collected, including 11 samples of adults with allergic rhinitis and 17 samples from individuals without underlying respiratory diseases.^18^ The workflow of this study is shown in (Fig. 1).

### Differential expression analysis

Raw microarray data were downloaded from the GEO database. Quality control was performed by visualizing boxplots, density plots, and principal component analysis (PCA) to detect potential outliers and batch effects. The data were then background-corrected and normalized using the Robust Multi-array Average (RMA) method implemented in the R/Bioconductor software package *limma*.^19^ Low-expression probes were filtered out to reduce noise and improve analysis accuracy. Differential expression analysis between AR and control samples was carried out using *limma*, with initial criteria of |logFC| > 0.5 and *P*-value <0.05. Statistical significance was further assessed using moderated t-tests, and multiple testing correction was applied via the Benjamini-Hochberg false discovery rate (FDR) method. Genes with an adjusted p-value (FDR) less than 0.05 were considered significantly differentially expressed. The results were visualized through heatmaps and volcano plots to highlight the significantly upregulated and downregulated genes.

### Weighted gene co-expression network analysis (WGCNA)

WGCNA^20^ was performed to identify gene modules correlated with AR. First, to reduce noise and focus on biologically relevant genes, we selected the top 50% of genes ranked by variance across samples. This filtering step is commonly used to improve network detection sensitivity. Second, quality control was conducted by removing genes and samples with excessive missing values or low expression levels to ensure data reliability. Third, the soft-thresholding power (β) was chosen based on the criterion that the scale-free topology fit index reached 0.85 for the first time, ensuring that the constructed network approximates a scale-free topology. This thresholding was used to calculate the adjacency matrix representing gene co-expression similarity. Fourth, genes were clustered into modules using average linkage hierarchical clustering based on topological overlap, and each module was assigned a unique color. Finally, gene dendrograms and module-trait relationships were visualized to interpret the results.

### GO and KEGG pathway enrichment analysis

GO and KEGG pathway enrichment analyses were performed using the Xiantao bioinformatics platform (https://www.xiantaozi.com/products/apply). This platform provides an integrated, user-friendly interface for comprehensive functional enrichment analysis, enabling efficient identification of significantly enriched biological processes and pathways. Although the website is primarily in Chinese, it offers robust analytical tools widely used in related studies, facilitating streamlined data analysis.^21^, ^22^, ^23^

### Machine learning

Five machine learning algorithms—LASSO, SVM-RFE, Boruta, XGBoost, and random forest—were applied to identify key genes associated with AR diagnosis. LASSO regression was implemented using the glmnet R package.^24^ The random forest model was developed using the randomForest R package,^25^ commonly used for classification and feature selection. For SVM-RFE, Boruta, and XGBoost, the respective R packages e1071,^26^ Boruta,^27^ and xgboost^28^ were utilized. The intersecting genes identified by all 5 algorithms were selected as candidate biomarkers.

### Immune infiltration analysis

The CIBERSORT algorithm^29^ was used to estimate the relative proportions of 22 immune cell subtypes in the nasal epithelial brushing samples from GSE19187, Immune cell abundance was visualized using heatmaps generated by the “corrplot” package.^30^

### Recipient operating characteristics curve (ROC) and area under the curve (AUC)

To evaluate the diagnostic performance of the identified feature genes for AR, ROC curve analysis was performed, and AUC values were calculated using the “pROC” R package. The analysis was conducted for both the training dataset (GSE19187) and the external validation dataset (GSE46171) to assess the sensitivity and specificity of each gene in distinguishing AR patients from healthy controls.

### Real-time quantitative PCR for validation

NP69 cells, an immortalized human nasopharyngeal epithelial cell line sourced from Vigen Biotechnology (Zhenjiang, China), were utilized to validate gene expression changes in vitro.^31^ The cells were treated with 5 μg/mL House Dust Mite (HDM, *Dermatophagoides pteronyssinus*) extract (Cat. No. XPB91D3A2.5, Greer Laboratories, USA) for 24 h, a condition optimized through preliminary experiments evaluating epithelial cytokine responses. Total RNA was isolated and purified using the FastPure Cell/Tissue Total RNA Isolation Kit V2 (Vazyme, RC112-01), and reverse transcribed into cDNA using the HiScript II One Step RT-PCR Kit (Vazyme, P611-01). Real-time quantitative PCR (RT-qPCR) was performed on an Applied Biosystems QuantStudio 5 system. Relative gene expression levels were calculated using the 2ˆ-ΔΔCt method. Statistical analyses and *P*-values were obtained using Prism GraphPad (version 8.0). The primer sequences were as follows: CST1: Forward 5′-TGGCCCAGTATCTGAGTACCCT-3′, Reverse 5′-CCGCAGCGGACGT CTGTAGT-3′; CST2: Forward 5′-GGGCCGAACCATATGTACCA-3′, Reverse 5′-GCAGTTCTG GCTGTTCATGGA-3′; SERPINB4: Forward 5′-CCACTGATGCATA TGAGCTGAAG-3′, Reverse 5′-CTGGTAAAATTTCTTGATGGCATCT-3′; TOX: Forward 5′-AAGGGCCAAAATCCAAACG-3′, Reverse 5′-CCCACATTG AAGCCA CAATTTTA-3’.

### Statistical analysis

All statistical analyses were performed using R software (version 4.2.1) and the Xiantao platform. Normality of continuous variables was assessed by the Shapiro–Wilk test. For comparisons between 2 groups, independent Student's t-tests were applied to data meeting normality assumptions; for comparisons among multiple groups, one-way ANOVA was used. When data did not meet normality criteria, appropriate non-parametric tests were applied. Multiple comparisons were corrected using the Benjamini–Hochberg false discovery rate method where applicable. Statistical significance was defined as *P*-value <0.05.

## Results

### Identification of DEGs

**Fig. 2:**
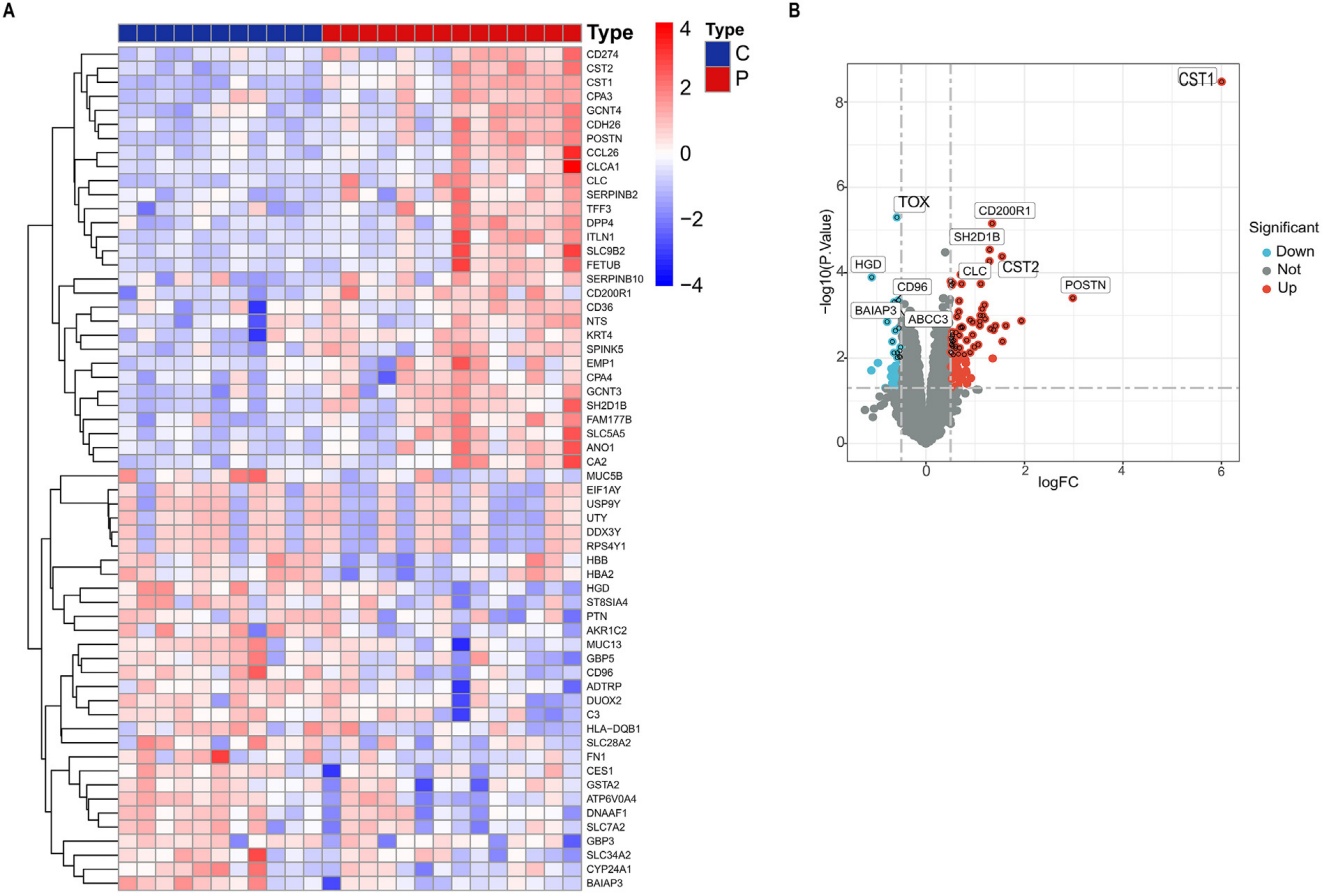
**The heat map and volcano map of the DEG identified in the AR dataset GSE19187**. (**A**) Each row represents DEG, and each column represents each sample of healthy or AR cases. Red and blue represent upregulated and downregulated DEGs, respectively. (**B**) The red and blue circles refer to DEGs with upregulated and downregulated gene expression, respectively. AR, Allergic rhinitis; DEGs, differentially expressed genes.

We identified a total of 133 DEGs between AR patients and healthy controls from the GSE19187 dataset using the limma package with multiple testing correction. Applying a p value of <0.05, 87 genes were significantly upregulated and 46 genes were significantly downregulated in AR patients compared to controls. The expression patterns of these DEGs are shown in the heatmap and volcano plot (Fig. 2A and B).

### WGCNA analysis and hub module selection

**Fig. 3:**
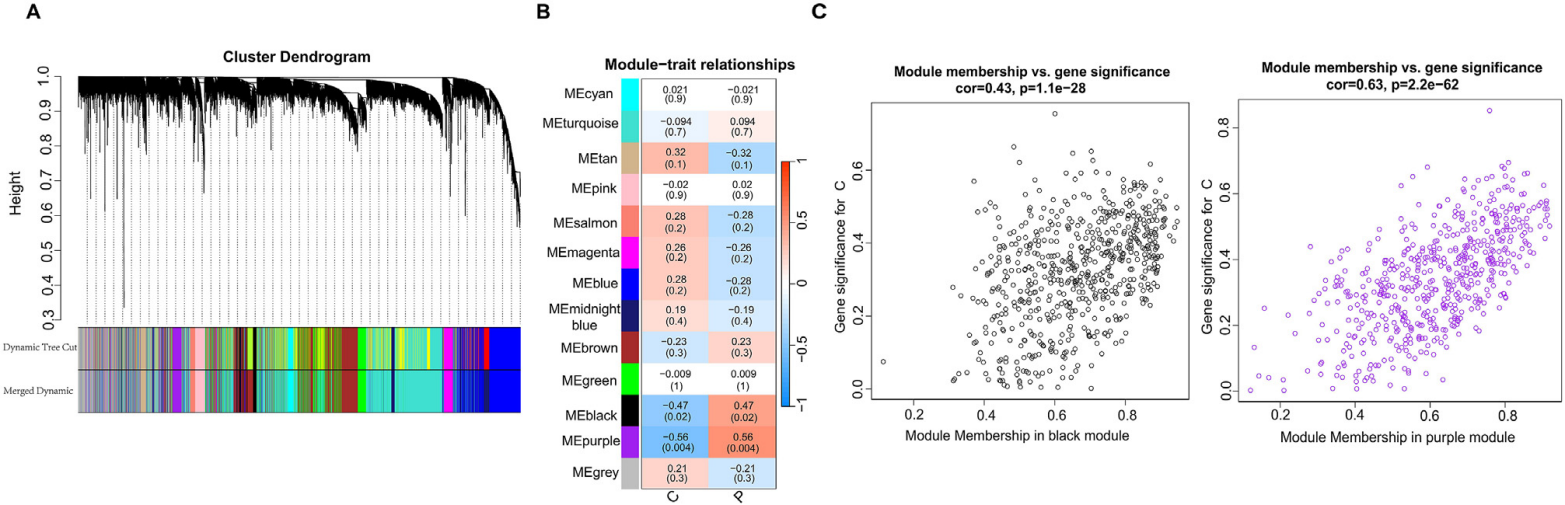
**The number of modules and genes from WGCNA.** (**A**) Cluster tree diagram of genes in GSE19187, where each branch represents a gene and each color represents a co-expressed module. (**B**) The correlation of co-expressed module genes obtained by WGCNA and the numbers in parentheses represent the *P-value* of genes in each module. WGCNA, Weighted gene co-expression network analysis. C, Control; P, Patients. (**C**) Scatter plot of gene significance (GS) between weight and module member relationship (MM) in black and purple modules. In this module, there is a highly significant correlation between GS and MM.

The flow diagram of our sample clustering dendrogram from WGCNA is presented in (SFig. 1A). To analyze the gene expression patterns of multiple samples, we clustered 20159 genes and analyzed the association between modules and specific traits or phenotypes from GSE19187. As illustrated in (SFig. 1B and C), the soft power threshold we set to 11 based on scale-free *Rˆ*2 = 0.9. Totally 13 modules were identified, which were the strongly correlated modules based on a 0.25 clustering height limit (Fig. 3A). From the selected modules, the black (R = 0.47, P = 0.02) and purple (R = 0.56, P = 0.004) modules showed moderate but statistically significant correlations with AR (Fig. 3B and C). These 2 modules had the highest module-trait correlation coefficients in our analysis. A total of 1158 candidate genes from the black and purple modules were included in subsequent analyses (Table S1).

### KEGG and GO enrichment analyses of overlapping DEGs

**Fig. 4:**
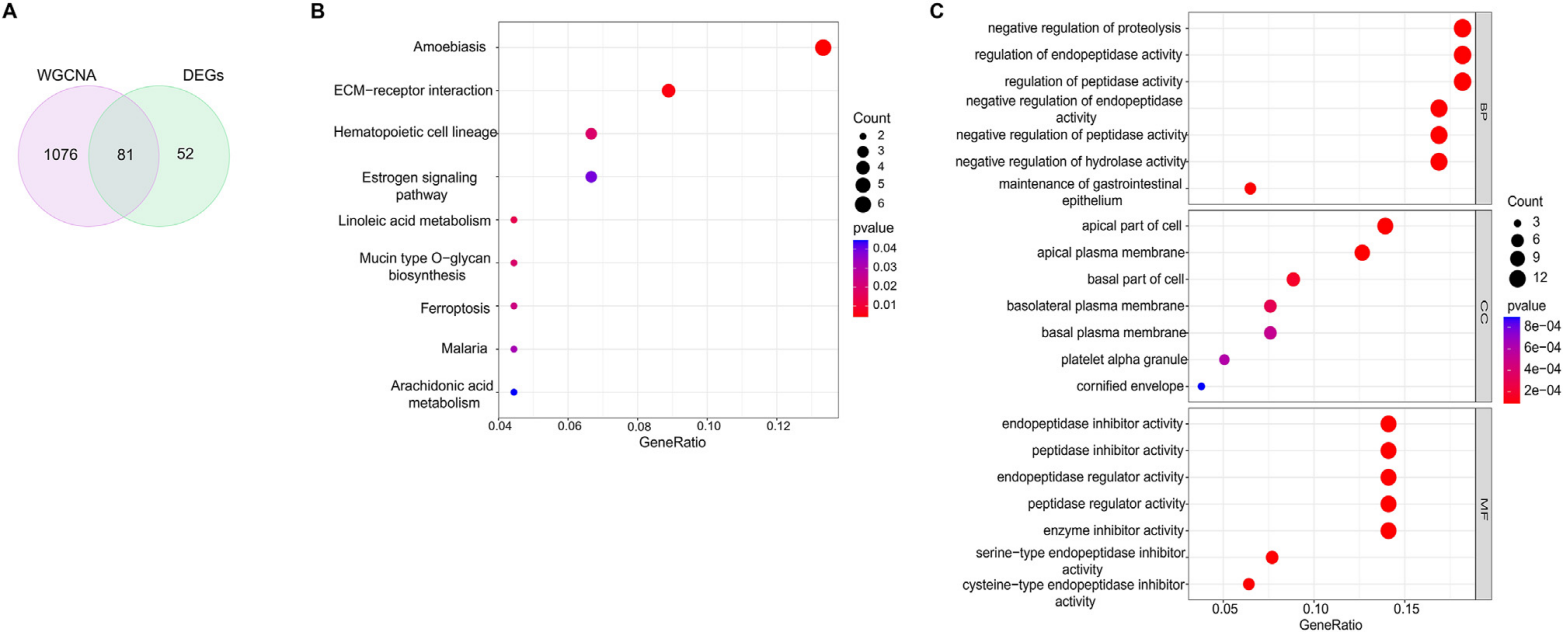
**Enrichment analysis of gene intersections in AR.** (**A**) The Venn plot shows that 81 genes were identified at the intersection of DEGs using black and purple module genes from WGCNA. (**B**) KEGG pathway analysis of gene crossover. Different colors represent various important pathways and related enriched genes. (**C**) GO analysis of gene crossover, including biological processes, cellular components, and molecular functions. The y-axis represents different GO terms, the x-axis represents the proportion of genes enriched in relative GO terms, the size of the circle represents the number of genes, and the color represents the *P-value*. AR, Allergic rhinitis. KEGG, Kyoto Encyclopedia of Genes and Genomes. GO, Gene ontology; WGCNA, Weighted gene co-expression network analysis; DEGs, Differentially Expressed Genes.

To analyze the biological features of the key gene sets, we identified 81 overlapping genes by intersecting the DEGs obtained from the limma analysis with the hub genes derived from the black and purple modules identified through WGCNA (Fig. 4A). Additionally, these genes were classified into amoebiasis, ECM (Extracellular matrix)-receptor interaction, hematopoietic cell lineage, estrogen signaling pathway, linoleic acid metabolism, mucin-type o-glycan biosynthesis, ferroptosis, malaria, and arachidonic acid metabolism in the KEGG and GO enrichment analysis (Fig. 4B and C), and enriched in cellular component terms, including the apical part of the cell, apical plasma membrane, basal part of the cell, basolateral plasma membrane, basal plasma membrane, platelet alpha granule, and cornified envelope (Fig. 4C). Regarding the molecular function terms, genes were mainly enriched in endopeptidase inhibitor activity, endopeptidase regulator activity, peptidase inhibitor activity, enzyme inhibitor activity, peptidase regulator activity, serine-type endopeptidase inhibitor activity, and cysteine-type endopeptidase inhibitor activity (Fig. 4C). The pathway analysis results were involved in AR development. The number of genes associated with ECM-receptor interaction occurred in the KEGG pathway analysis, suggested a higher correlation with AR.

### Four feature genes identified by integrating 5 machine-learning algorithms

**Fig. 5:**
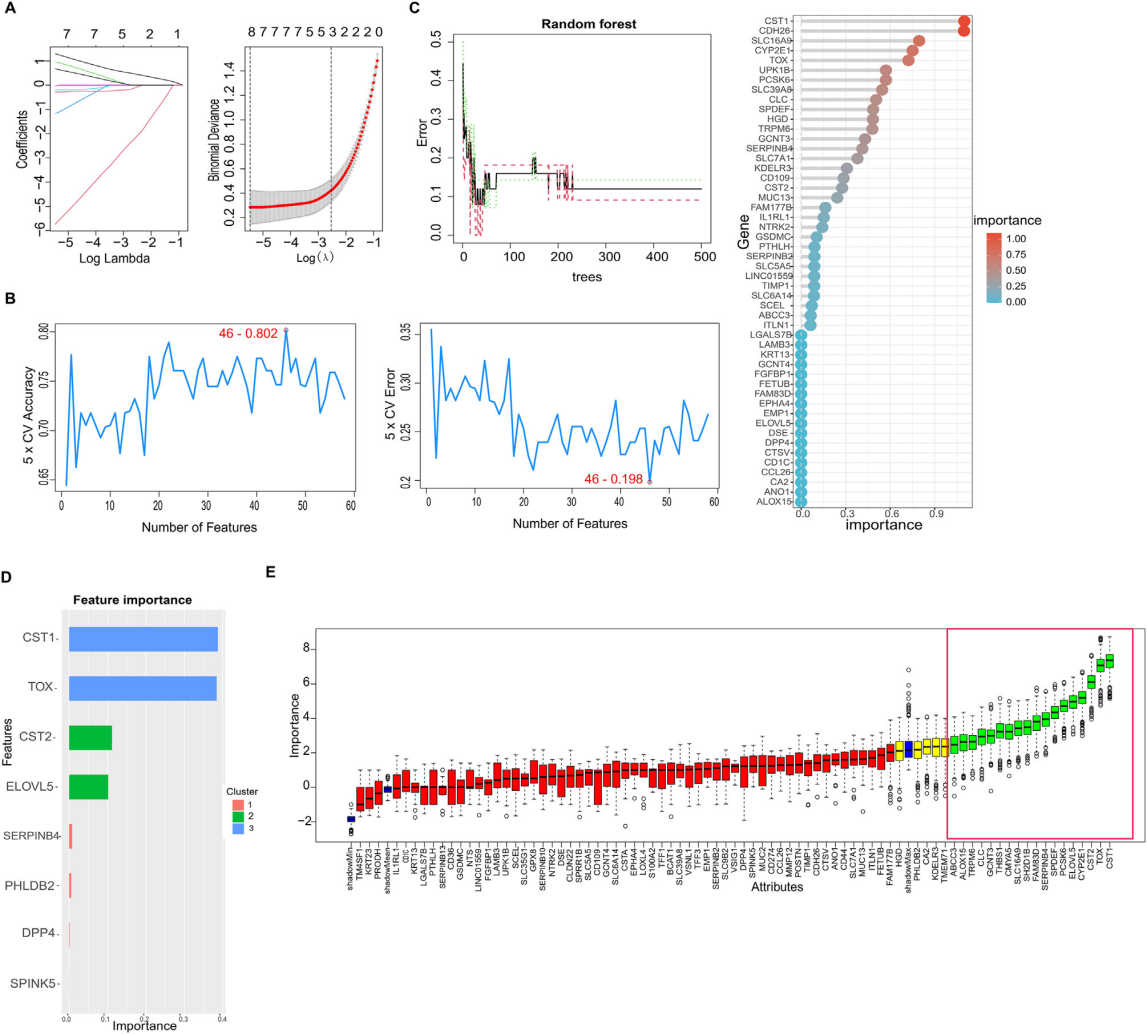
**Five machine learning methods for identifying the best central genes.** (**A**) Screening of biomarkers in the LASSO model. Coefficient distribution map, where 1 line represents genes and the vertical axis represents coefficients. The solid line in the left figure represents partial likelihood deviation, and the number of genes corresponding to the lowest point of cure (n = 81) is most suitable for LASSO. The vertical axis of the right panel represents the binary deviation, which can be understood as the size of the model error. The left row displays the smallest error, while the right row displays fewer features. (**B**) The SVM-RFE algorithm is used to further select the best candidate genes, which is based on the highest accuracy and lowest error obtained from the curve. The x-axis displays the number of feature selections, while the y-axis displays the prediction accuracy. (**C**) The random forest algorithm displays the errors in AR; Sort the control group and genes based on importance scores. Use random forest to calculate the relative importance of overlapping candidate genes (importance>0.2). The importance index is plotted on the x-axis, while genetic variables are plotted on the y-axis. (**D**) The characteristic importance of factors predicting mortality in AR patients in XGBoost. (**E**) Boruta selected 84 feature genes with importance ranking. The green bar chart is considered meaningful.

**Fig. 6:**
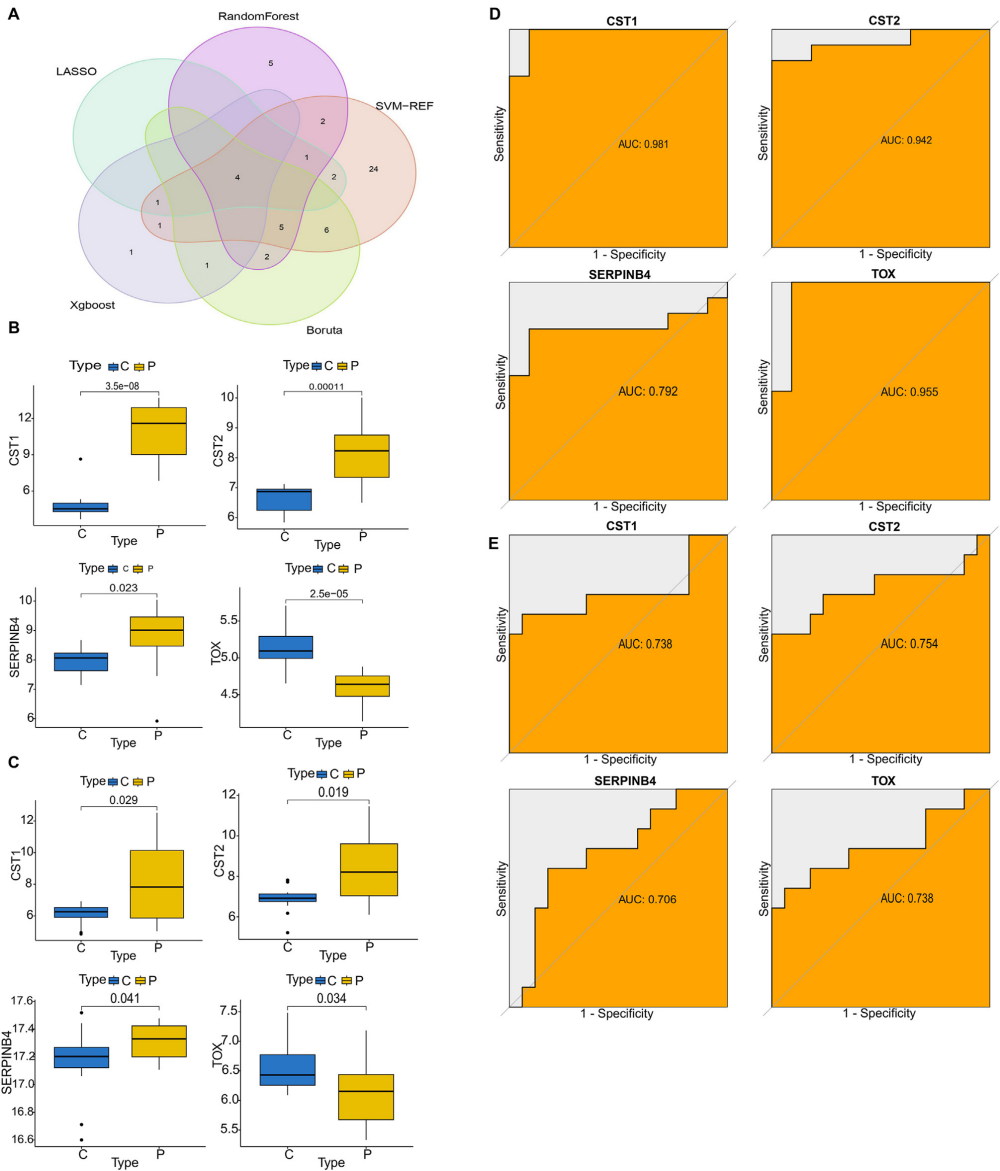
**The susceptibility genes of AR were identified.** (**A**) The flower chart shows the 4 best feature genes shared by LASSO: random forest, SVM-RFE algorithm, XGBoost, and Boruta algorithm. (**B**) The expression levels of CST1, CST2, SERPINB4, and TOX in GSE19187. (**C**) The expression levels of CST1, CST2, SERPINB4, and TOX in GSE46171. (**D**) The ROC curves of 4 genes serve as a gene set for AR patients in GSE19187. (**E**) The ROC curves of 4 genes in GSE46171. C, Control; P, Patients; AR, Allergic rhinitis; DEGs, Differentially expressed genes; ROC, Receiver operating characteristics. ∗ P < 0.05, ∗ ∗ P < 0.01 and ∗∗∗ P < 0.0001.

To improve the robustness of feature gene selection, we applied 5 machine learning methods—LASSO, SVM-RFE, Random Forest (RF), XGBoost, and Boruta—to the 81 candidate genes. LASSO identified 8 genes, SVM-RFE selected 46 genes, RF identified 19 genes with importance >0.2, XGBoost yielded 8 genes, and Boruta selected 18 genes (Fig. 5A–E, Table S2–S4). To enhance specificity and reduce potential overfitting, we focused on the intersection of genes consistently identified across all 5 methods, resulting in 4 common genes: CST1, CST2, SERPINB4, and TOX (Fig. 6A). These genes were subsequently validated in an independent dataset (GSE18574), where they maintained consistent expression patterns and diagnostic potential (Fig. 6B and C), supporting the robustness and reproducibility of this integrative approach.

### Verification of the expression and diagnostic significance of characteristic genes

The expression levels of the 4 feature genes were checked in 14 AR samples and 11 healthy samples in GSE19187. While CST1, CST2, and SERPINB4 were upregulated in AR, TOX was decreased in this disease. Moreover, the same changes of 4 genes were verified in 11 patients with AR and 17 healthy individuals in GSE46171 (Fig. 6A–C; P < 0.05). Furthermore, ROC curve analysis was conducted to quantitatively assess the diagnostic and predictive value of the characteristic genes (Fig. 6D and E). The AUC value of CST1 was 0.981, CST2 was 0.942, SERPINB4 was 0.792, and TOX was 0.955 in GSE19187 (Fig. 6D). Simultaneously, the external validation dataset CSE46171 presented high AUC values: CST1 (AUC:0.738), CST2 (AUC:0.754), SERPINB4 (AUC:0.706), and TOX (AUC:0.738), demonstrating that these hub feature genes predicted progression and exhibited a convincing diagnostic value for AR (Fig. 6E).

### Immune cell infiltration of characteristic genes

**Fig. 7:**
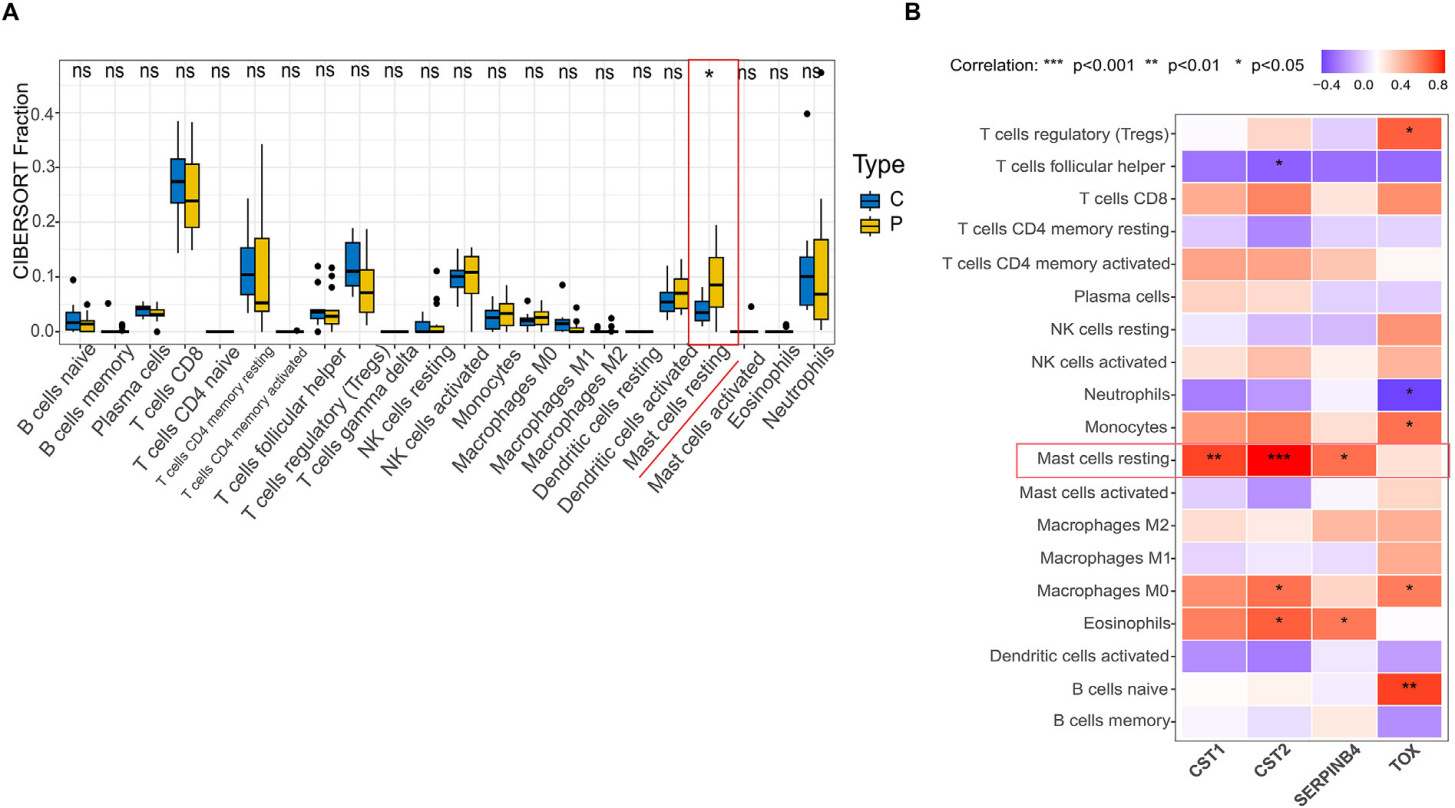
**Analysis of immune cell infiltration between control and AR.** (**A**) The proportion of 22 types of immune cells in different samples can be seen from the box diagram. (**B**) Comparing the *P*-values of 19 immune cells between the AR group and the control group through a correlation matrix diagram. ∗*P* < 0.05, ∗ ∗ *P* < 0.01 and ∗∗∗ *P* < 0.001. The vertical axis displays subtypes of immune cells. AR, Allergic rhinitis.

Immune infiltration analysis was conducted using the CIBERSORT algorithm to investigate the role of four genes in immune regulation. The graphs for differential immune infiltration are revealed in (Fig. 7A and B). Compared with control samples, the proportion of resting mast cells was upregulated in AR samples, while the others demonstrated no significant difference. Additionally, the above 4 genes showed greater differences in immune infiltration analysis. While CST1, CST2, and SERPINB4 showed higher expression in resting mast cells, TOX exhibited no significant difference in this analysis. Overall, 4 genes screened from immune infiltration may be potential regulators for AR treatment.

### QPCR for mRNA levels of predicted hub genes

**Fig. 8:**
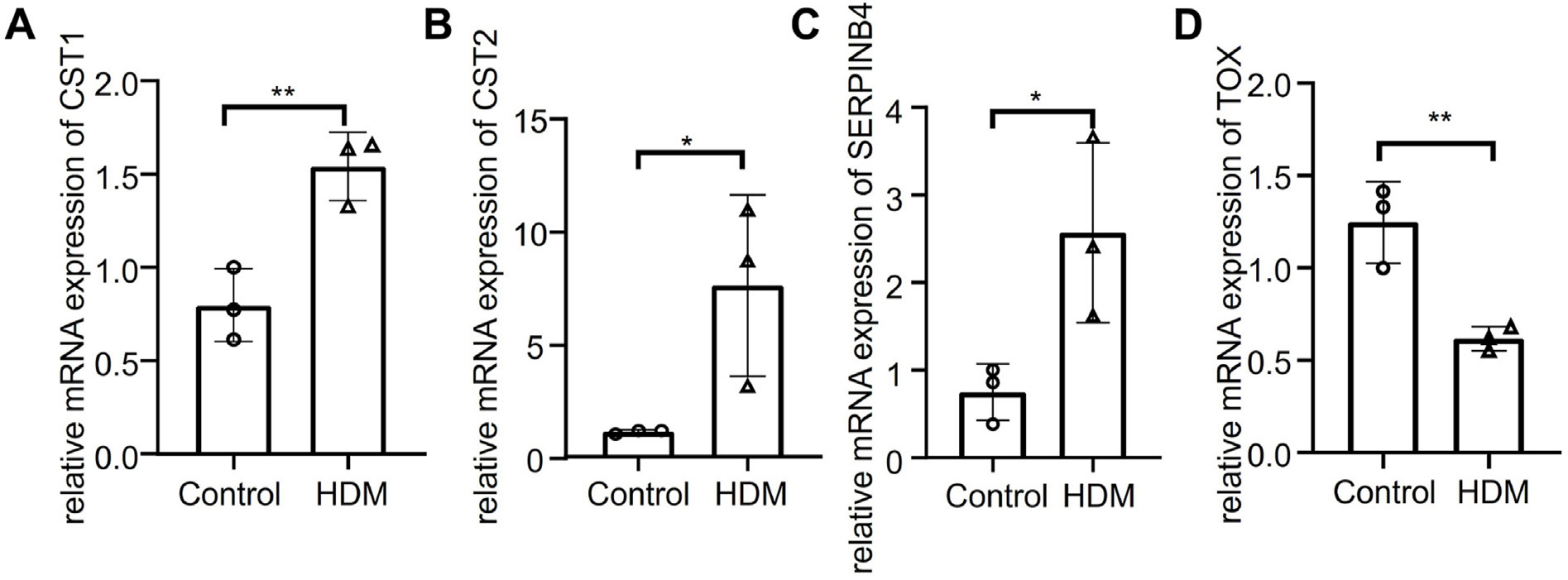
**Validation of diagnostic gene expression by RT-qPCR.** (A–D) The expressions of (**A**) CST1. (**B**) CST2. (**C**) SERPINB4. (**D**) TOX. between 5 μg/mL HDM-treatment NP69 cell line and Control subjects. ∗*P <* 0.05 and ∗∗*P <* 0.01.

To verify differential expressions of CST1, CST2, SERPINB4, and TOX, the human nasopharyngeal epithelial cell line (NP69) was used to assess mRNA expression levels of these genes via Real-time quantitative PCR. The expression trends of CST1, CST2, and SERPINB4 followed public databases, and the expression of TOX was lower in the AR group (Fig. 8A–D). Consequently, the changes in the 4 gene expressions did not differ in the common stable cell line, suggesting that the 4 genes were of high clinical diagnostic value.

## Discussion

AR is a prevalent chronic inflammatory condition of the nasal mucosa, triggered by IgE-mediated reactions to allergens such as pollen, dust mites, and animal dander.^32^ This condition significantly impairs the quality of life, leading to symptoms such as nasal congestion, rhinorrhea, sneezing, and itching, which can further result in sleep disturbances, fatigue, and decreased cognitive function.^33^ The global burden of AR is substantial, affecting approximately 10–30% of the population, with increasing prevalence in urban areas.^34^ Despite its high prevalence and impact, the underlying molecular mechanisms of AR remain incompletely understood, necessitating further research to develop more effective diagnostic and therapeutic strategies.

In this study, we focused on the nasal epithelial transcriptome to elucidate the detailed mechanisms of AR, leveraging advanced bioinformatics and machine learning techniques. The nasal epithelium is the primary site of allergen exposure and immune response initiation, making it a critical area for understanding AR pathophysiology.^35^ By analyzing gene expression profiles from publicly available datasets (GSE19187 and GSE46171), we identified DEGs and constructed gene co-expression networks to uncover key molecular pathways involved in AR. Our integrative approach, combining differential expression analysis, WGCNA, and multiple machine learning algorithms, has the potential to identify novel biomarkers and therapeutic targets for AR. The identification of key diagnostic genes and the development of predictive models could significantly enhance the accuracy of AR diagnosis and pave the way for personalized treatment strategies.^36^ Using 5 machine learning analyses and CIBERSORT to infer specific immune cell infiltration, we found that the expression status of 3 genes (CST1, CST2, and SERPINB4) was highly correlated with resting mast cell infiltration, except for TOX, which is associated with naive B cells.

As members of the type 2 cystatin (CST) superfamily, CST1(Cystatin SN) and CST2(Cystatin SA) have been reported to be capable of inhibiting the proteolytic activities of cysteine proteases and is implicated in progression of several human cancers.^37^^,^^38^

CST1 has been implicated in various inflammatory and immune responses. Recent studies have shown that CST1 is significantly upregulated in patients with AR, suggesting its role in the pathogenesis of this condition.^39^ The elevated expression of CST1 in AR patients may contribute to the modulation of protease activity, thereby influencing the inflammatory milieu in the nasal epithelium. NP-69 cell line is a classic nasal epithelial cell used to study nasopharyngeal related cancers. Our study corroborates these findings, demonstrating a marked increase in CST1 expression in AR samples compared to controls. This upregulation was further validated through qPCR in NP69 cells treated with HDM extract, reinforcing the potential of CST1 as a diagnostic biomarker for AR. Therefore, CST1 not only serves as a critical marker for AR diagnosis but also provides insights into the molecular mechanisms underlying the disease.

Similar to CST1, CST2 has been found to be upregulated in allergic conditions, including AR.^40^ Our analysis identified CST2 as one of the keys differentially expressed genes in AR, with significant upregulation observed in both the primary dataset and external validation dataset. The qPCR results in NP69 cells further confirmed this trend, highlighting CST2's potential as a diagnostic marker. The role of CST2 in modulating protease activity and its impact on immune cell function, particularly in the context of mast cell activation, aligns with the observed immune cell infiltration patterns in AR.^40^ These findings suggest that CST2 may play a crucial role in the pathophysiology of AR, making it a valuable target for further research and potential therapeutic intervention. It is well known that serine protease inhibitors could be one of the treatments for inflammation.

SERPINB4, also known as squamous cell carcinoma antigen 2, is a member of the serpin family of protease inhibitors.^41^ This gene has been associated with various inflammatory and immune responses, particularly in epithelial tissues.^42^ Our study identified SERPINB4 as one of the key genes upregulated in AR patients. The increased expression of SERPINB4 in AR samples, validated through qPCR in NP69 cells, suggests its involvement in the inflammatory process's characteristic of AR. The role of SERPINB4 in inhibiting serine proteases may contribute to the regulation of protease activity and the maintenance of epithelial integrity in the nasal mucosa.^42^ Additionally, the association of SERPINB4 with immune cell infiltration, particularly mast cells, underscores its significance in the immune response observed in AR. These findings highlight the potential of SERPINB4 as a diagnostic biomarker and a therapeutic target for AR, providing new avenues for understanding the molecular mechanisms underlying the disease.

TOX, or thymocyte selection-associated high mobility group box protein, is a transcription factor involved in the regulation of T-cell development and function, and is associated with thymocyte selection, is expressed differently in all stages of T lymphocyte development.^43^ TOX is a nuclear deoxyribonucleic acid-binding factor^44^ that induces a set of chemokine genes in immune cells and modulates inflammation through the production of interleukin-10.^45^ However, TOX has not been previously associated with AR, suggesting it may be a valuable potential research target. Unlike the other identified genes, TOX was found to be downregulated in AR patients. This downregulation was consistent across both the primary and validation datasets, as well as in qPCR validation using NP69 cells. The decreased expression of TOX in AR suggests a potential role in modulating immune responses, particularly in the context of T-cell function and differentiation. These findings indicate that TOX may play a protective role in the immune response associated with AR, and its downregulation could be a contributing factor to the disease's pathogenesis. Further research is needed to elucidate the precise mechanisms by which TOX influences AR and to explore its potential as a therapeutic target.

It is interesting that 3 of the 4 genes are associated with protease. There was other evidence that ADAM10 and ADAM17 influenced allergic rhinitis,^46^ which are also transmembrane metalloproteases. It is well known that serine protease inhibitors were selected as treatments for inflammation, because proteases’ dysregulation may induce severe consequences, like inflammation of neurology, skin, lung and arthritis.^47^ It could be predicted that the inhibition of proteases may be a promising target of AR.

The enrichment analysis results from our study provide significant insights into the biological processes and pathways involved in allergic rhinitis (AR). KEGG pathway analysis revealed that the differentially expressed genes (DEGs) are involved in pathways such as amoebiasis, ECM-receptor interaction, hematopoietic cell lineage, and estrogen signaling pathway. These pathways are crucial for understanding the underlying mechanisms of AR. For instance, the ECM-receptor interaction pathway is essential for cell adhesion and communication, which are critical in inflammatory responses and tissue remodeling observed in AR.^48^ The hematopoietic cell lineage pathway is involved in the development and differentiation of immune cells, which are pivotal in the immune response associated with AR.^49^ Additionally, the estrogen signaling pathway has been implicated in modulating immune responses and inflammation, suggesting a potential link between hormonal regulation and AR pathogenesis.^50^

GO enrichment analysis further highlighted the involvement of DEGs in biological processes such as the negative regulation of proteolysis and regulation of endopeptidase activity. These processes are important for maintaining protease-antiprotease balance, which is often disrupted in inflammatory conditions like AR.^51^ The cellular component analysis indicated significant enrichment in apical and basal plasma membranes, suggesting that these genes may play roles in maintaining epithelial barrier integrity and function, which is often compromised in AR.^52^

In conclusion, the enriched pathways and biological processes identified in this study provide a comprehensive understanding of the molecular mechanisms underlying AR. Future studies should focus on further elucidating the roles of these pathways and genes in AR, and collecting peripheral blood samples from patients to validate the diagnostic value of these 4 genes in large samples, in order to enhance our understanding and management of this condition.

The immune infiltration analysis conducted using the CIBERSORT algorithm revealed significant differences in immune cell subtype proportions between healthy and AR groups, highlighting the role of immune cells in the pathogenesis of AR. Specifically, the analysis identified an increased proportion of resting mast cells in AR samples compared to controls. Mast cells are known to play a crucial role in allergic reactions by releasing histamine and other mediators that contribute to inflammation and symptom manifestation in AR patients.^53^ The elevated expression of CST1, CST2, and SERPINB4 in resting mast cells suggests that these genes may be involved in the regulation of mast cell activity and, consequently, in the allergic response observed in AR.

Certainly, our study has several limitations, such as using only ROC curves to predict the value of susceptible genes, small sample sizes, lack of validation cohorts, and absence of AR severity data, which may affect our conclusions.

## Conclusion

In summary, this study successfully identified differentially expressed genes associated with allergic rhinitis and visualized these findings through heatmaps and volcano plots. WGCNA analysis revealed gene modules closely related to AR, and GO and KEGG enrichment analyses highlighted key biological processes and pathways involved in AR mechanisms. Five machine learning algorithms identified 4 key diagnostic genes, and a predictive model was developed. The CIBERSORT algorithm demonstrated significant differences in immune cell subtype proportions between healthy and AR groups. The ROC curve and AUC values indicated high accuracy of the classification model. Finally, real-time quantitative PCR validated the differential expression of key genes in the NP69 cell line. These findings provide valuable insights into the molecular mechanisms of AR and offer potential biomarkers for diagnosis and therapeutic targets. Future studies should aim to address the current limitations by incorporating larger sample sizes, clinical validation, and wet lab experiments to further substantiate these findings.

## Abbreviations

AR, allergic rhinitis; CD109, C3 And PZP-Like Alpha-2-Macroglobulin Domain-Containing Protein 7; CD14, lipopolysaccharide receptor; CDH26, cadherin-Like Protein 26; CLC, charcot-Leyden Crystal Protein; CST1, cystain-SA-I; CST2, cystatin 2; CYP2E1, cytochrome P450 Family 2 Subfamily E Member 1’GCNT3, glucosaminyl (N-Acetyl) Transferase 3; GEO, gene Expression Omnibus; HGD, homogentisate 1,2-Dioxygenase; KDELR3, KDEL Endoplasmic Reticulum Protein Retention Receptor 3; LMRGs, lipid metabolism-related genes; PCSK6, proprotein Convertase Subtilisin/Kexin Type 6; SERPINB4, serpin Family B Member 4; SLC16A9, solute Carrier Family 16 Member 9; SLC39A8, solute Carrier Family 39 Member 8; SLC7A1, solute Carrier Family 7 Member 1;SPDEF, SAM Pointed Domain Containing ETS Transcription Factor; STAT1, signal transducer and activator of transcription 1; TLR4 , toll-like receptor 4; TOX , thymocyte Selection Associated High Mobility Group Box; TRPM6, transient Receptor Potential Cation Channel Subfamily Member 6; UPK1B, uroplakin 1B; WGCNA, weighted gene co-expression network analysis

## Availability of data and materials

The datasets analyzed during the current study are available in the Gene Expression Omnibus (GEO) website, numbers were GSE19187 and GSE46171.

## Author contributions

HL and YC designed the study. QC, JF and YL analyzed the data and wrote the primary manuscript. QC and JF prepared the figures and tables from the literature. All authors reviewed and approved the final manuscript.

## Ethics approval and consent to participate

Not applicable.

## Authors' consent for publication

We guarantee our manuscript is original, has not been published before, is not currently being considered for publication elsewhere, and has not been posted to a preprint server. The publication is approved by all authors and that, if accepted, it will not be published elsewhere in the same form, in English or in any other language, without the written consent of the copyright-holder.

## Funding

This study was supported by the Taihu Lake talent plan (Top-Level, no. 2020THRC-GD-7), the 333 project of 10.13039/501100002949Jiangsu Province in 2022 (ZUZHIBU 202221001).

## Declaration of competing interest

The authors declare that they have no competing interests.

## Figures and Tables

**Supplementary Figure 1:**
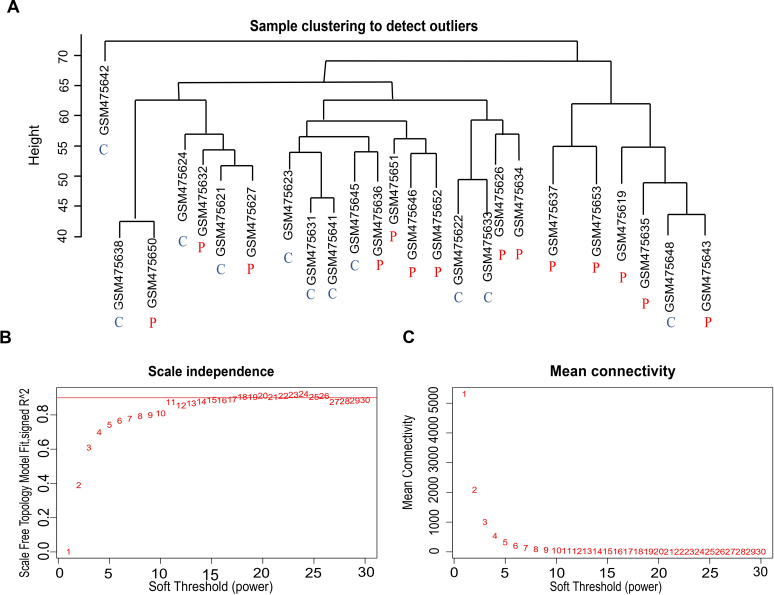
WGCNA processing of AR dataset GSE19187. (A) Cluster tree diagram of samples from WGCNA. (B–C) The power value of the adjacency matrix in WGCNA, where the red line represents 0.9 on the vertical axis. AR, Allergic rhinitis; WGCNA, Weighted gene co expression network analysis.

## Appendix A Supplementary data

Supplementary Table S1 Raw data of weighted correlation network analysis (WGCNA). Supplementary Table S1

Supplementary Table S2 The 81 candidate genes identified from the LASSO analysis. Supplementary Table S2

Supplementary Table S3 The 46 feature genes were screened from the SVM-RFE algorithm. Supplementary Table S3

Supplementary Table S4 Eight feature genes through Xgboost algorithms and 18 hub genes from Boruta. Supplementary Table S4

## References

[1] Nur Husna S.M., Tan H.T., Md Shukri N., Mohd Ashari N.S., Wong K.K. (2022). Allergic rhinitis: a clinical and pathophysiological overview. Front Med (Lausanne).

[2] Pfaar O, Angier E, Muraro A, Halken S, Roberts G (2020). Algorithms in allergen immunotherapy in allergic rhinoconjunctivitis. Allergy.

[3] Ma Z, Teng Y, Liu X (2017). Identification and functional profiling of differentially expressed long non-coding RNAs in nasal mucosa with allergic rhinitis. Tohoku J Exp Med.

[4] Seo JH, Kim HY, Jung YH (2015). Interactions between innate immunity genes and early-life risk factors in allergic rhinitis. Allergy Asthma Immunol Res.

[5] Lei Y, Guo P, An J (2018). Identification of pathogenic genes and upstream regulators in allergic rhinitis. Int J Pediatr Otorhinolaryngol.

[6] Wang Y, Qi X, Li H (2025). Inhibition of TRPV1 attenuates innate nasal epithelial responses via NF-kappaB signaling pathway in allergic rhinitis. Int Immunopharmacol.

[7] Sha J, Yang M, Lei Y (2025). Interaction between nasal epithelial cells and tregs in allergic rhinitis responses to allergen via CCL1/CCR8. Front Immunol.

[8] Hu W, Xie F, Wu Y (2025). Identification and validation of key amino acids in IgE linear epitopes of beta-Lactoglobulin: comparison of recognition patterns of Chinese bovine milk-allergic sera with different symptoms. J Agric Food Chem.

[9] Su W, Tian Y, Wei Y, Hao F, Ji J (2023). Key genes and immune infiltration in chronic spontaneous urticaria: a study of bioinformatics and systems biology. Front Immunol.

[10] Wen S, Li F, Tang Y (2023). MIR222HG attenuates macrophage M2 polarization and allergic inflammation in allergic rhinitis by targeting the miR146a-5p/TRAF6/NF-kappaB axis. Front Immunol.

[11] Zhou S, Zhou C, Wang X (2023). Profiles of immune infiltration in seasonal allergic rhinitis and related genes and pathways. Int Immunopharmacol.

[12] Zhu G, Cai H, Ye L (2021). Small proline-rich protein 3 regulates IL-33/ILC2 axis to promote allergic airway inflammation. Front Immunol.

[13] Nie J, Jiang X, Wang G (2024). Yu-Ping-Feng-San alleviates inflammation in atopic dermatitis mice by TLR4/MyD88/NF-kappaB pathway. J Ethnopharmacol.

[14] Costa-Pinto F.A., Basso A.S. (2012). Neural and behavioral correlates of food allergy. Chem Immunol Allergy.

[15] Xue K, Yang J, Zhao Y, Cheng J, Wang Z (2020). Identification of susceptibility genes to allergic rhinitis by gene expression data sets. Clin Transl Sci.

[16] Wang Y, Wang J, Yan Z, Liu S, Xu W (2023). Microenvironment modulation by key regulators of RNA N6-methyladenosine modification in respiratory allergic diseases. BMC Pulm Med.

[17] Kimura H, Francisco D, Conway M (2020). Type 2 inflammation modulates ACE2 and TMPRSS2 in airway epithelial cells. J Allergy Clin Immunol.

[18] McErlean P, Berdnikovs S, Favoreto S (2014). Asthmatics with exacerbation during acute respiratory illness exhibit unique transcriptional signatures within the nasal mucosa. Genome Med.

[19] Ritchie ME, Phipson B, Wu D (2015). Limma powers differential expression analyses for RNA-sequencing and microarray studies. Nucleic Acids Res.

[20] Langfelder P., Horvath S. (2008). WGCNA: an R package for weighted correlation network analysis. BMC Bioinf.

[21] Chen N., Luan Y. (2025). Specific expression and common potential therapeutic drugs in different brain regions of major depressive disorder patients: bioinformatics analysis. J Affect Disord.

[22] Wang Y, Huang Y, Zhu H (2024). Exostoisns (EXT1/2) in head and neck cancers: an in silico analysis and clinical correlates. Int Dent J.

[23] Zhu Y, Chen Y, Xie D (2023). Macrophages depletion alleviates lung injury by modulating AKT3/GXP4 following ventilator associated pneumonia. Front Immunol.

[24] Trevor Hastie, B.E., Lars: least angle regression, lasso and forward stagewise*.* 2022.

[25] Liaw A W.M. (2002). Classification and regression by randomForest. R News.

[26] (2024). e1071: Misc functions of the department of statistics, probability theory group (formerly: E1071), TU Wien.

[27] Kursa M.B., R.W (2010). Feature selection with the boruta package. J Stat Software.

[28] Chen T., G.C., XGBoost (2016).

[29] Newman AM, Liu CL, Green MR (2015). Robust enumeration of cell subsets from tissue expression profiles. Nat Methods.

[30] Salome P.A., Merchant S.S. (2021). Co-expression networks in chlamydomonas reveal significant rhythmicity in batch cultures and empower gene function discovery. Plant Cell.

[31] Xu J, Li J, Wang X (2025). IRF4 knockdown inhibits the chronic rhinosinusitis without nasal polyps development by regulating NLRP3/Caspase-1/GSDMD-Mediated pyroptosis. Biochem Genet.

[32] Bousquet J, Khaltaev N, Cruz AA (2008). Allergic rhinitis and its impact on asthma (ARIA) 2008 update (in collaboration with the world health organization, GA(2)LEN and AllerGen). Allergy.

[33] Meltzer E.O. (2016). Allergic rhinitis: burden of illness, quality of life, comorbidities, and control. Immunol Allergy Clin North Am.

[34] Pawankar R, Canonica GW, Holgate ST, Lockey RF (2012). Allergic diseases and asthma: a major global health concern. Curr Opin Allergy Clin Immunol.

[35] Baroody F.M. (2003). Allergic rhinitis: broader disease effects and implications for management. Otolaryngol Head Neck Surg.

[36] Zhang L, Han D, Huang D (2009). Prevalence of self-reported allergic rhinitis in eleven major cities in China. Int Arch Allergy Immunol.

[37] Dai DN, Li Y, Chen B (2017). Elevated expression of CST1 promotes breast cancer progression and predicts a poor prognosis. J Mol Med (Berl).

[38] Ou R., Lin C., Chen Y. (2023). CST2 is activated by RUNX1 and promotes pancreatic cancer progression by activating PI3K/AKT pathway. Arch Biochem Biophys.

[39] Wang M, Gong L, Luo Y (2023). Transcriptomic analysis of asthma and allergic rhinitis reveals CST1 as a biomarker of unified airways. Front Immunol.

[40] Xu Z, Forno E, Sun Y (2024). Nasal epithelial gene expression and total IgE in children and adolescents with asthma. J Allergy Clin Immunol.

[41] Sivaprasad U, Kinker KG, Ericksen MB (2015). SERPINB3/B4 contributes to early inflammation and barrier dysfunction in an experimental murine model of atopic dermatitis. J Invest Dermatol.

[42] Bu X, Wang M, Yuan J (2024). SerpinB3/B4 abates epithelial cell-derived CXCL8/IL-8 expression in chronic rhinosinusitis with nasal polyps. J Immunol Res.

[43] Niu H., Wang H. (2023). TOX regulates T lymphocytes differentiation and its function in tumor. Front Immunol.

[44] O'Flaherty E., Kaye J. (2003). TOX defines a conserved subfamily of HMG-box proteins. BMC Genom.

[45] Canaria DA, Rodriguez JA, Wang L (2023). Tox induces T cell IL-10 production in a BATF-dependent manner. Front Immunol.

[46] Chueh HW, Park SK, Hur DY, Bae WY (2015). Expression profile of ADAM10 and ADAM17 in allergic rhinitis. Int Forum Allergy Rhinol.

[47] Soualmia F., El Amri C. (2018). Serine protease inhibitors to treat inflammation: a patent review (2011-2016). Expert Opin Ther Pat.

[48] Hynes R.O. (2009). The extracellular matrix: not just pretty fibrils. Science.

[49] Orkin S.H., Zon L.I. (2008). Hematopoiesis: an evolving paradigm for stem cell biology. Cell.

[50] Straub R.H. (2007). The complex role of estrogens in inflammation. Endocr Rev.

[51] Lopez-Otin C., Bond J.S. (2008). Proteases: multifunctional enzymes in life and disease. J Biol Chem.

[52] Guttman-Yassky E., Krueger J.G. (2017). Atopic dermatitis and psoriasis: two different immune diseases or one spectrum?. Curr Opin Immunol.

[53] Galli S.J., Tsai M., Piliponsky A.M. (2008). The development of allergic inflammation. Nature.

